# A comparison of the chemo- and radiotoxicity of thorium and uranium at different enrichment grades

**DOI:** 10.1007/s00204-023-03484-6

**Published:** 2023-04-06

**Authors:** A. Rump, C. Hermann, A. Lamkowski, T. Popp, M. Port

**Affiliations:** Bundeswehr Institute of Radiobiology, Neuherberg Str. 11, 80937 Munich, Germany

**Keywords:** Uranium, Thorium chemotoxicity, Radiotoxicity, Stochastic radiation effects, Deterministic radiation effects

## Abstract

Uranium and thorium are heavy metals, and all of their isotopes are radioactive, so it is impossible to study chemical effects entirely independent of the radiation effects. In the present study, we tried to compare the chemo- and radiotoxicity of both metals, taking into account deterministic radiation damages reflected by acute radiation sickness and stochastic radiation damages leading to long-term health impairments (e.g., tumor induction). We made at first a literature search on acute median lethal doses that may be expected to be caused by chemical effects, as even acute radiation sickness as a manifestation of acute radiotoxicity occurs with latency. By simulations based on the biokinetic models of the International Commission on Radiological Protection and using the Integrated Modules for Bioassay Analysis software, we determined the amounts of uranium at different enrichment grades and thorium-232 leading to a short-term red bone marrow equivalent dose of 3.5 Sv considered to cause 50% lethality in humans. Different intake pathways for incorporation were considered, and values were compared to the mean lethal doses by chemotoxicity. To assess stochastic radiotoxicity, we calculated the uranium and thorium amounts leading to a committed effective dose of 200 mSv that is often considered critical. Mean lethal values for uranium and thorium are in the same order of magnitude so that the data do not give evidence for substantial differences in acute chemical toxicity. When comparing radiotoxicity, the reference units (activity in Bq or weight in g) must always be taken into account. The mean lethal equivalent dose to the red bone marrow of 3.5 Sv is reached by lower activities of thorium compared to uranium in soluble compounds. However, for uranium as well as thorium-232, acute radiation sickness is expected only after incorporation of amounts exceeding the mean lethal doses by chemotoxicity. Thus, acute radiation sickness is not a relevant clinical issue for either metal. Concerning stochastic radiation damages, thorium-232 is more radiotoxic than uranium if incorporating the same activities. Using weight units for comparison show that for soluble compounds, thorium-232 is more radiotoxic than low-enriched uranium in the case of ingestion but even more toxic than high-enriched uranium after inhalation or intravenous administration. For insoluble compounds, the situation differs as the stochastic radiotoxicity of thorium-232 ranges between depleted and natural uranium. For acute effects, the chemotoxicity of uranium, even at high enrichment grades, as well as thorium-232 exceeds deterministic radiotoxicity. Simulations show that thorium-232 is more radiotoxic than uranium expressed in activity units. If the comparison is based on weight units, the rankings depend on the uranium enrichment grades and the route of intake.

## Introduction

Uranium and thorium are actinoids, a group of chemical elements with atomic numbers ranging from 89 (actinium) to 103 (lawrencium), including well-known radionuclides like plutonium or americium (Katz et al. 2006). All actinoids are heavy metals and radioactive. In contrast to other representatives of the group, both uranium and thorium are found in the earth's crust, thorium being twice–three times more common than uranium. Only three isotopes of uranium are found naturally (uranium-234, -235, and -238) (Grenthe et al. 2006). In the case of thorium, only one isotope, thorium-232, is present in appreciable amounts in nature (Wickleder et al. 2006; Ménétrier et al. 2005).

The main civilian and military uses of uranium and thorium are shown in Table 1. Because of its fissile nature and its ability to sustain chain reactions, uranium-235 is used as fuel in nuclear reactors at natural or low enrichment grades (few percent) as well as for the construction of nuclear weapons at high enrichment grades (> 90%). The uranium-235 enrichment process produces a residuum with a lower weight percentage of uranium-235 (depleted uranium, DU). In civilian settings, this depleted uranium is used as counterweights and ballast in aircrafts and ships as well as in the production of special steels as alloying material. In weapon systems, depleted uranium is used in kinetic armor-piercing ammunitions as well as to armor combat vehicles (Sztajnkrycer and Otten 2004). Depleted uranium piercing ammunitions were in particular used during the Gulf war (1991) and in Kosovo (1999), and suspicions of harmful health effects for the engaged military personnel and the local population were raised in the media (Katz 2014). Many reports in the press linked depleted uranium exposure to the Gulf War syndrome and leukemia in Balkans peacekeeping forces, although no evidence for this relation could be found (Roth et al. 2001; Sztajnkrycer and Otten 2004). For the sake of completeness, it should be mentioned that in the past, uranium compounds were also used in ‘colors’ for ceramics and for some time even in the treatment of diabetes (Kathren 2009), which contributed to our knowledge of the toxicity of uranium in men.

**Table 1:** Main civilian and military use of uranium and thorium compounds

Uranium	Thorium
Civilian use	
Fuel in nuclear reactors	Possible fuel in nuclear reactors
Counterweights and ballast in aircrafts and ships (DU)	Light source in gas mantles ("Welsbach mantle", “Auerlicht.”)
Alloying material in special steels	Alloying elements in welding electrodes, glasses including camera and scientific instrument lenses
Treatment of diabetes (historical)	Medical radiographic contrast product (Thorotrast) (historical)
Military use	
Kinetic armor-piercing ammunition (DU)	Alloys for electronic and aeronautic equipments in military aircraft
Armor in combat vehicles (DU)	Encapsulated infrared heat radiator in guided missiles

Thorium is used in various industries too (Leiterer et al. 2010; McDiarmid et al. 2012): it has been widely used in the preparation of gas lantern mantles, in high-intensity discharge lamps to optimize lamp stability and lifetime, as an additive to glass in optical instruments, as a chemical catalyst or to manufacture thoriated tungsten electrodes. Thorium-232, as fertile material, can principally also be used as a fuel in nuclear reactors via the absorption of a slow neutron and generation of the fissionable isotope uranium-233 (secondary nuclear fuel). However, this application is not used at a commercial level for the time being (Wickleder et al. 2006). A well-known medical application was its use in medicine as a radiographic contrast product (commercial names, e.g., Thorotrast^®^) mainly used to visualize vascular structures (ATDSR 2019). Another less-known medical application for some time (1944–1951) was the use of thorium compounds for the treatment of tuberculosis or spondylarthritis ankylosans (Paquet 2014). Among its industrial use, thorium is included in alloys (e.g., with magnesium), particularly for electronic and aeronautic equipment. For example, the J79 turbojet engine used in various fighter and bomber aircraft (e.g., F-104 G Starfighter or F-4 Phantom) contains large parts of thorium–magnesium alloys (Kuipers and Schirmer 2019). Although thorium-232 is an alpha emitter, and alpha and beta decay dominate in its decay chain, some members of the chain also emit gamma-radiation (Laroche 1998; Ménétrier et al. 2005). It could, however, be shown that in Air Force maintenance and repair facilities, the emanating dose rates from gamma-radiation do not represent a health hazard in realistic, conservative scenarios (effective dose < 1 mSv/year, dose limit for skin not exceeded) (Kuipers and Schirmer 2019). Workers on the ATAR turbojet assembly line have been reported being exposed to an annual dose of 1.8 to 4.2 mSv (Laroche et al. n.d.) which is still below the limits permitted by European occupational regulations (20 mSv).

A less well-known application of thorium-232 is its use in some anti-tank guided missiles, e.g., the Milan missile (Missile ‘d’infanterie léger antichar). In this device, the trajectory control, which is considered very reliable, takes place via a steering wire, and the optical tracking by the gunner is done by an infrared tail, which comes about by an incandescent lamp in the missile also containing thorium-232 (as far as publicly known, about 10 kBq, i.e., 10,000 Bq/4060 Bq/g = 2.5 g) (Hofmann 2001; BMVg 2014). The radioactive material is released during the flight and at the time of detonation on the target with the generation of radioactive dust (IPPNW 2014). Thus, military training areas or combat zones where these weapon systems are used might become radioactively contaminated (Laquai 2014).

Uranium, as well as thorium are both heavy metals, and all of their nuclides are radioactive. Thus, uranium and thorium must be considered radiotoxic, with the radiation hazard resulting not solely but mainly from alpha-radiation (Sztajnkrycer and Otten 2004). In the case of uranium, besides animal studies, research efforts were highly intensified during the Manhattan project in the 1940s, and data were later compiled in the United States Transuranium and Uranium Registries (USTUR) that was established in 1978 (Kathren 2009). Although the radiotoxic effects of uranium are often in the focus of the public fear, numerous experimental studies and clinical cases showed that uranium nephrotoxicity caused by chemotoxic mechanisms is much more pronounced than its radiotoxicity (Rump et al. 2019). The toxicity of thorium has, in particular, been investigated regarding its use in intravenously administered radiological contrast media containing the metal in colloidal form (e.g., Thorotrast, Umbrathor, Toriofanino) (Mori et al. 1999; Becker et al. 2008; Ménétrier et al. 2005; ATDSR 2019). These preparations were widely used in the period between 1930 and 1950. Besides granuloma at the injection site, hepatic tumors (hemangioendothelioma, hemangiosarcoma, cholangiosarcoma, hepatic carcinoma) were observed decades later. Thorium in colloidal form injected intravenously is avidly taken up into the reticuloendothelial system (mainly in the liver), with an inhomogeneous distribution and a large variability, whereas the amounts retained in the red bone marrow and the bone have been reported as relatively low (Kaul and Muth 1978). Thus, colloidal thorium preparations have particular pharmacokinetic properties, and the findings on colloidal thorium contrast agents cannot just be transferred to other inorganic thorium compounds (ATDSR 2019). The toxicity of the latter is much less investigated, and experimental data are in part quite ancient, particularly concerning acute toxicity. Although thorium is considered more radiotoxic than uranium in occupational medicine, this relates to stochastic radiation damages (probability of the occurrence of long-term health effects like tumors) (Prise and O’Sullivan 2009) that must be clearly differentiated from deterministic radiation damages (induction of acute radiation sickness) and chemotoxicity as well.

Because uranium and thorium are used in weapon systems, both radioactive metals are important for military health services to properly assess and to avoid health hazards emanating from exposure and correctly judge on possible justified or unjustified compensation claims by servicemen. The present study aims to compare the chemo- and radiotoxic properties of uranium and thorium taking also into account different uranium enrichment grades.

## Method

### Literature search and comparison of acute toxicity data for uranium and thorium

To compare the chemical toxicity of uranium and thorium, we made a literature search on acute poisonings in humans and lethal doses (preferably mean lethal doses, LD_50_) in animals. As we are interested in the chemical toxicity at this stage, we focused on acute toxicity of single doses to reasonably exclude lethal deterministic radiation damages occurring with latency. Moreover, we particularly considered animal experiments with parenteral administration of the chemicals to determine the toxicity of systemically available uranium or thorium and exclude the modifying effects caused by variable absorption fractions. This seems particularly important as even soluble uranium and thorium compounds are reported to possess a low bioavailability in the case of ingestion or inhalation. Thus, only small absolute variations in the absorption quote may lead to large relative changes in bioavailability and toxic effects, making quantitative comparisons more difficult.

### The biokinetic-dosimetric models used for radiological dose assessments after uranium or thorium incorporation

**Fig. 1:**
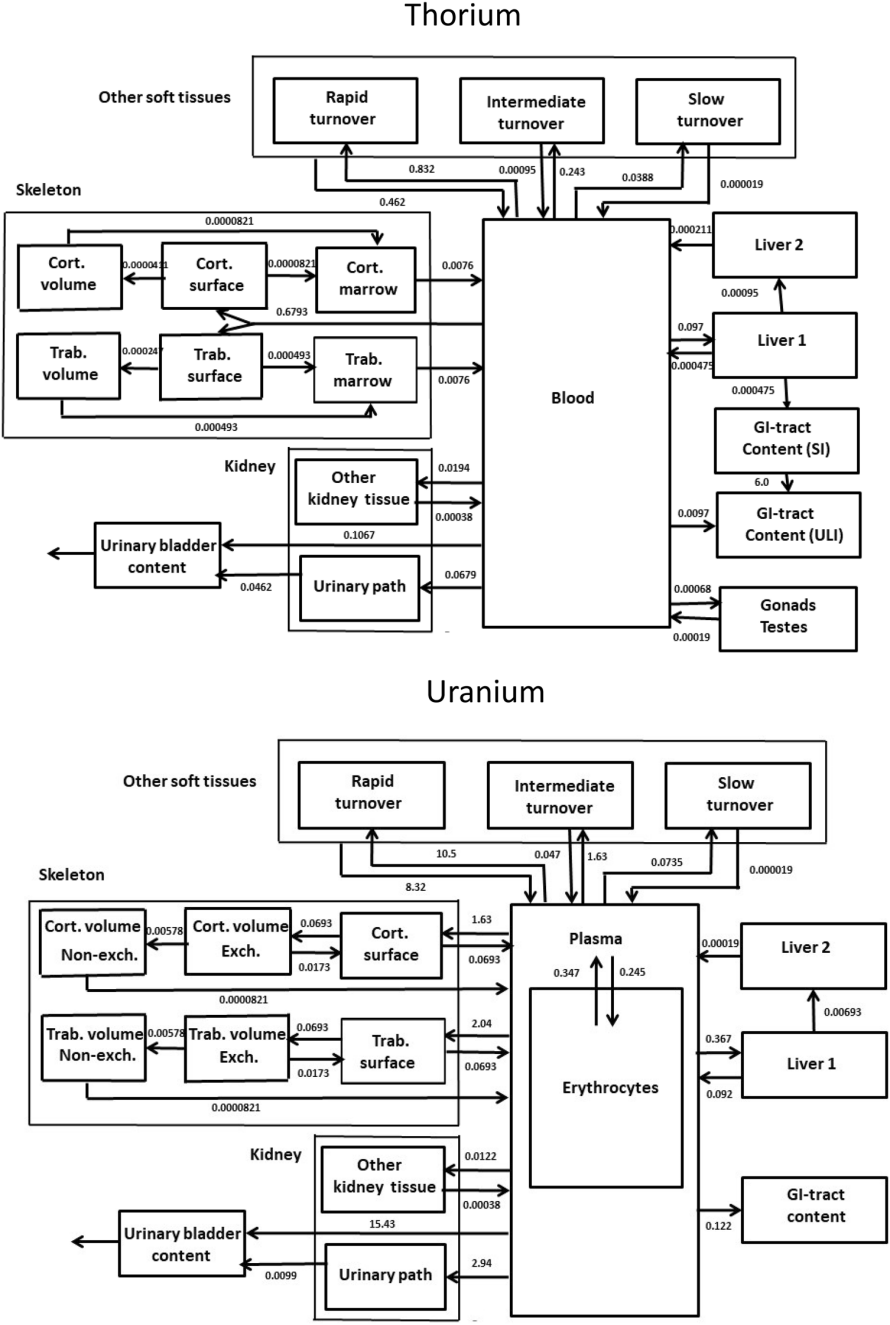
The ICRP systemic models for uranium and thorium (Source: ICRP 1995a, 1997). *Cort*. Cortical, *Trab*. trabecular, *Exch*. exchangeable, *GI* gastrointestinal, *SI* small intestine, *ULI* upper large intestine. The numbers on the arrows are the rate (transfer) constants in day^−1^. The rate constant from the transport from SI to ULI (6 day^−1^) in the thorium model is from the ICRP gastrointestinal model (ICRP 1979). Note in particular the different structures of the skeleton tissue

The assessment of radiotoxicity is made particularly difficult in so far as the absorbed radiological doses after radionuclide incorporation cannot be directly measured by physical means but only computed based on biokinetic and dosimetric models (Rump et al. 2016). Therefore, the validity and reliability of results heavily depend on the structure and parameters of these models based on human and experimental animal data, as far as available. All our estimations of radiological doses were calculated using the commercial internal dosimetry software package “Integrated Modules for Bioassay Analysis” (IMBA Professional Plus Version 4.0) (James et al. 2005; Birchall et al. 2007). The calculations are based on the generic non-radionuclide specific models of the International Commission on Radiological Protection (ICRP) for inhalation (ICRP 1994a, 1994b) and ingestion (ICRP 1979), and the specific systemic biokinetic models for uranium or thorium (ICRP 1995a, 1997) (Fig. 1). The structure of the biokinetic model for earth-alkaline metals is applied to uranium, and the model for actinides to thorium (with specific parameters for each metal). The models differ in particular in the structure of the bone tissue (ICRP 1995a, 1997; Chevalier et al. 1997). As route of intake, we considered the administration of soluble compounds by intravenous injection, ingestion, or inhalation (type “F” compounds, absorption fraction for adults *f* = 0.02 and 0.0005 for uranium and thorium, respectively) (ICRP 1995b) For thorium, we also considered the exposure to a poorly soluble compound by inhalation to take into account the hazards associated to the spread of thorium-232 by the detonation of an anti-tank missile (type “S” compound, *f* = 0.0005 similar to “F” compounds, but slower dissolution rates in the inhalational absorption model). In the latter case, we considered only stochastic radiation damages, as deterministic effects are expected to be much smaller due to the very low absorption quotes. In all cases, the following assumptions were made for inhaled particles: sigma-G 2.4977, density 3 g/ml, shape 1.5, activity median aerodynamic diameter (AMAD) of 5 µm) (default values of IMBA).

For practical purposes, it is justified to consider only the decay of a parent nuclide when the daughter nuclide is stable or when the first progeny radionuclide has a very long half-life, and the in-growth of this progeny can be neglected (progeny rule by IMBA: “truncation”). Otherwise, the in-growth of the progeny has to be taken into account (progeny rule by IMBA: “super merge”). If the progeny radionuclide has a very short physical half-life (i.e., < 0.01 day), the emitted radiation and energy can just be added to the emission of the parent (progeny rule by IMBA: “merge”).

**Fig. 2:**
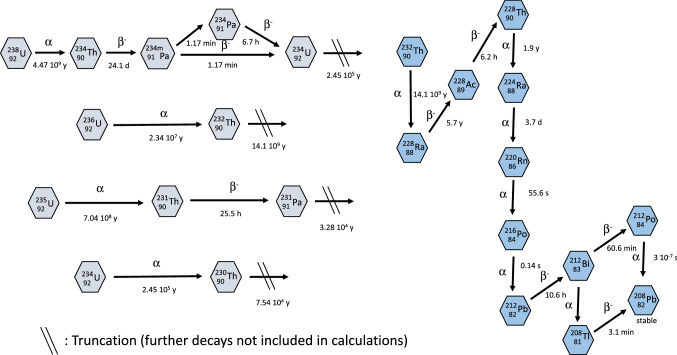
The decays of the decay chain of uranium-235, uranium-238 and thorium-232 included for the calculation of the radiological doses by IMBA ("super merger" principles). The decay of further progeny in the decay chain of uranium-isotopes is not taken into account in the computations ("truncation"). U-234. The additional emission of photons (e.g., gamma-radiation) is not included in the indications of decay. Nuclear decay data from ICRP (2008)

The decay of uranium-238 leads to thorium-234, which has short-lived progeny (protactinium-234 m and protactinium-234) whose decay results in the formation of uranium-234 with a long radioactive half-life. Thus, radionuclide decays following the formation of uranium-234 are not entered into the computations (Fig. 2) (nuclear decay data from ICRP 2008). In the case of uranium-235, the decay of thorium-231 (half-life 26 h) to protactinium-231 was considered in addition to the decay of the parent nuclide. For uranium-236, only the initial decay was considered as it decays to thorium-232 with a very long half-life (1.4 × 10^10^ a). The same applies to uranium-234, giving birth to thorium-230 (7.54 × 10^4^ a). In both cases, IMBA applies the truncation progeny rule.

In our simulations, we considered natural, depleted as well as low (3.5%), and highly enriched (93.5%) uranium using the isotopic composition of the IMBA software database (Table 2). Uranium-236 is absent in natural uranium. It is generated by the capture of a neutron by uranium-235. Its presence is considered as evidence that the material originates at least partly from the reprocessing of spent nuclear fuel (Desideri et al. 2002; Sztajnkrycer and Otten 2004). As uranium-236 has been identified in very low amounts in depleted uranium products (US-EPA 2006), it was included in our computations.

**Table 2:** Specific activity and isotopic composition of natural, depleted, and enriched uranium mixtures. Source: IMBA database

	U-234	U-235	U-236	U-238
Depleted uranium (14,879 Bq/g)				
Mass (%)	0.001	0.2	0.0003	99.8
Activity (%)	15.46	1.07	0.05	83.42
Natural uranium (25,270 Bq/g)				
Mass (%)	0.0054	0.720	0	99.274
Activity (%)	48.9	2.3	0	48.9
Slightly enriched uranium (81,460 Bq/g)				
Mass (%)	0.03	3.5	0	96.4
Activity (%)	81.8	3.4	0	14.7
Highly enriched uranium (2,520,100 Bq/g)				
Mass (%)	1.06	93.5	0.21	5.3
Activity (%)	96.8	2.97	0.2	0.03

Uranium-236 is a “man-made” isotope found in spent fuel or reprocessed uranium. Its presence may be considered as evidence that the sample has been in a nuclear reactor

In the case of thorium-232, the whole decay chain was taken into account with seven intermediate half-life progeny (228Ra, 228Ac, 228Th, 224Ra, 212Pb, 212Bi, 208Tl) (“super merge”) and direct “mergers” of all shorter-lived progeny (220Rn, 216Po, 212Po) with their respective parents (Fig. 2).

This also corresponds to the German Radiation Protection Ordinance, which, when setting limit values for certain radionuclides (e.g., exemption limits or limit values for surface contamination) (Annex 4, Table 1), also includes the exposure resulting from the decay of the daughter nuclides, as listed in Annex 4, Table 2 of the ordinance (StrlSchV 2018).

### Deterministic radiation toxicity: quantitative comparison of the theoretical median lethal doses based on radiotoxicity

The equivalent dose is the energy dose (energy absorbed by a mass unit of tissue) multiplied by a quality factor representing the relative biological effect of the particular radiation. The relative biological effect is an experimentally determined value for a defined endpoint. Although of factor of 20 is usually given for alpha particles, this value should be applied only for stochastic radiation effects (Sgouros et al. 2010; Lassmann and Nosske 2013). In our simulations of deterministic effects, we applied a lower factor of 5 as Lassmann and Nosske (2013) proposed. The equivalent dose permits to assess deterministic effects showing threshold values (e.g., acute radiation sickness to be expected at an acute whole-body dose exceeding 1 Sv) (Hall and Giaccia 2019). The most sensitive tissue is the red bone marrow that is depressed, leading to a hematopoietic syndrome, the first sub-syndrome of acute radiation sickness. In humans, hematological damages by radiation develop rather slowly compared to many animal species, with a peak incidence of death around the 30th day, and that’s why the median lethal radiological dose by a short-term whole-body irradiation causing death (bone marrow death) is given for the period within 60 days (LD_50/60_). The best estimate for this value in humans has been given at 3.5 Sv (Hall and Giaccia 2019). Using simulations with IMBA software, we determined the amounts of uranium or thorium (by injection, ingestion, or inhalation) necessary to deliver within the first 10 days an equivalent dose of 3.5 Sv. These amounts of uranium or thorium were considered to be the best estimate for the mean lethal doses based on the nuclide's acute radiotoxicity.

The IMBA software indicates the total radiological dose absorbed in the 50 years following incorporation. Still, it does not permit directly calculating the fraction of the dose absorbed at different time points. In analogy to methods applied in pharmacology and toxicology, we used the area under the activity time curve (AUC) as a metric reflecting the total dose (Derendorf and Garrett 1987). Departing from the total body activities given by IMBA for the first 200 days after radionuclide incorporation, we used the time-activity data points from day 100 to 200 to fit a mono-exponential equation. This function was used to simulate the activity time course on the right of the last time-activity value given by IMBA. As thorium is eliminated very slowly from the body, and we cannot just neglect the part of the curve for abscissa values over 50 years (time period for which the committed effective dose is calculated), we determined the area under the curve up to day 18,250 (50 years × 365 days) using the fitted exponential function and applying the trapezoidal rule for integration on time intervals of 10 days. Thus, the fraction of the AUC from day 0 to day 10 can be considered as the fraction absorbed during this period of the total dose absorbed up to 50 years after incorporation, as given by IMBA. It must be mentioned that this fraction based on the curve for the whole-body retention of activity was also used to quantitate the equivalent dose absorbed by the red bone marrow during the first 10 days after incorporation and this may impair the precision of our estimations.

To improve the comparison of the radiotoxicity of uranium and thorium, we calculated the ratios of the median lethal doses of thorium-232 and uranium at different enrichment grades using activity or weight units. We also determined the enrichment grade of uranium mixtures being toxicologically equivalent to thorium-232 based on weight units. The uranium-235 enrichment leads simultaneously to a percentage increase of uranium-234 in the mixture, whereby the enrichment of both isotopes is disproportionate. Uranium-234 cannot be neglected as it has a significantly higher specific activity than uranium-235 and 238, and therefore contributes significantly to the overall activity of the mixture even in natural uranium, despite a very low weight fraction (Table 2). To calculate the uranium-234 content in enriched uranium, the following formula of Rucker and Johnson (1997) was used:

$$\mathrm{\% }\left(\mathrm{weight}\right)\mathrm{U}-234\hspace{0.17em}=\hspace{0.17em}0.000054\times \left(\mathrm{\% U}-235\right)^2\hspace{0.17em}+\hspace{0.17em}0.0058\times \mathrm{\% U}-235\hspace{0.17em}+\hspace{0.17em}0.0015.$$

It must be pointed out that in enriched uranium, the ratio of uranium-234 and 235 can vary by more than 50%, so the results of our calculation results must be considered just as estimates. This is also reflected in the differences between the values calculated using the above formula and the composition as given by IMBA software (e.g., U-234 in depleted uranium: 0.001% vs. 0.0027%; highly enriched uranium: 1.016% vs. 1.06%).

In addition to the equivalent dose absorbed by the red bone marrow, we also computed the equivalent doses absorbed by other organs/tissues to visualize the different distributions of the radiological load for both uranium and thorium.

### Stochastic radiation toxicity: comparison of critical uranium or thorium amounts

Unlike from the previous section, we used a quality factor of 20 (and not 5) as a multiplier of the absorbed energy dose to derive the equivalent dose, as usual, for stochastic radiation damages. The effective dose is calculated by multiplying the equivalent doses to all tissues and organs specified by the ICRP, with radiation weighting factors characteristic for the sensitivity of the tissues to stochastic damages and summing up the values for all organs. The factors given by the International Commission on Radiological Protection were used (ICRP 1991, 1994). After radionuclide incorporation, it is usual to sum up the doses absorbed in a time frame of 50 years following the incident (70 years for children) (committed effective dose). The effective dose indicates stochastic health effects like, e.g., the occurrence of cancer. Averaged over both genders and all age groups, 1 mSv effective dose absorption is associated with a loss of a statistical lifetime of 0.4 days (Oka 2012; Rump et al. 2018a).

Irradiation enhances the probability of long-term health impairments, but there is no scientifically valid threshold value (linear non-threshold model) (Boice 2017). Nevertheless, permissible limits have been set up for occupational settings and the general public. In the case of a nuclear or radiological incident (e.g., “dirty bomb” attack) with the incorporation of radioactivity, it is generally considered that there is an indication for decorporation treatment if the committed effective dose exceeds 20–200 mSv (Ménétrier et al. 2005; Rump et al. 2018b).

We determined the amounts of uranium or thorium (by injection, ingestion, or inhalation) in soluble compounds leading to a committed effective dose of 200 mSv. These amounts of uranium or thorium were considered to be critical values in the case of a nuclear or radiological incident regarding stochastic radiation damages. As for the acute radiotoxicity, we also determined the ratios of the critical amounts of thorium-232 relative to uranium and the enrichment grade of uranium mixtures being equivalent to thorium-232 expressed in weight units regarding stochastic radiation effects.

In addition, we considered the inhalation of critical amounts of uranium or thorium included in poorly soluble compounds as would be expected in the case of oxides. Differences between uranium and thorium in the radiological loads of different tissues/organs were made evident. Although this is not a realistic scenario, but just a thought experiment, we also calculated the committed effective dose and the radiological doses absorbed by individual tissues/organs that would result from the inhalation of the whole radioactive load of a MILAN anti-tank missile (10 kBq).

## Results

### Comparison of the acute chemical toxicity of uranium and thorium

**Fig. 3:**
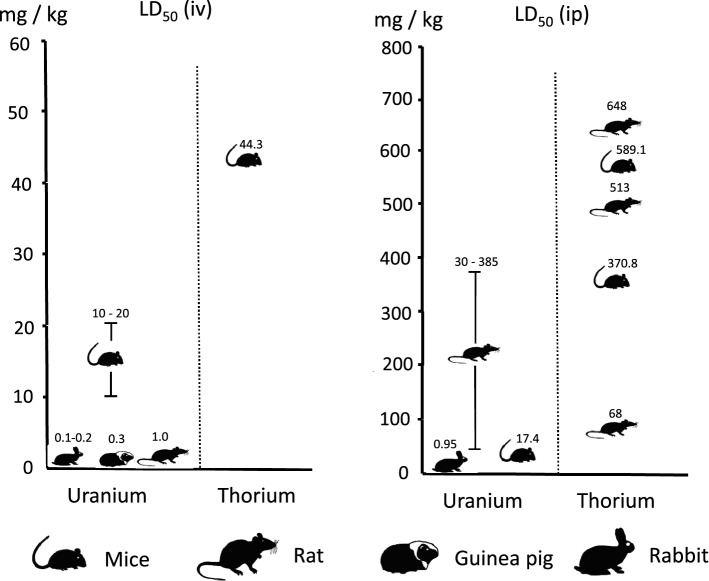
Median lethal doses of soluble uranium or thorium compounds after a single intravenous or intraperitoneal injection. Sources of the data: McClinton and Schubert (1948), Haven and Hodge (1949), Voegtlin and Hodge (1953), Watanabe (1957), Downs et al. (1959), Syao-Shan (1970), Kurlyandskaya (1970), McDiarmid et al. (2012) and Sangeetha Vijeyan et al. (2018)

The experimental median lethal doses of uranium in animals are species-dependent, and there is a decreasing sensitivity for uranium from rabbits (median lethal dose 0.1 mg/kg for iv administration), guinea pigs (0.3 mg/kg), rats (1 mg/kg) to mice (10–20 mg/kg) (Haven and Hodge 1949; Voegtlin and Hodge 1953; McDiarmid et al. 2012) (Fig. 3; Table 3). After intraperitoneal injection, the values reported as median lethal doses are in part definitely higher [e.g., the numerous values given by Haven and Hodge (1949) for rats, 30–385 mg/kg] or in a comparable range as for intravenous administration (mice LD_50_: 17.37 mg/kg, given by Sangetha Vijayan et al. 2018) (Table 3). For oral administration as a single dose, the mean lethal amounts were determined with 114 mg/kg in Sprague Dawley rats and with 136 mg/kg in Swiss-Webster mice (Domingo et al. 1987) and, thus, are in the same order of magnitude as some data for intraperitoneal injection. Lethal doses also seem to depend on the compounds used (toxicity order: uranyl fluoride > uranyl nitrate > uranyl chloride) and the age of the animals, e.g., young rats being more resistant than older rats (Haven and Hodge 1949) (Table 3).

**Table 3:** Median lethal doses by acute (chemo)toxicity of a single dose of uranium or thorium reported for different species depending on the route of intake.

Radionuclide	Species	Pathway	LD_50_ (mg/kg)	Source
Uranium	Rabbit	i.v	0.2^a^ (UO_2_(NO_3_)_2_·6H_2_O)	Hunter (1927)
	Rabbit	i.v	0.1 (UO_2_(NO_3_)_2_·6H_2_O)	Haven and Hodge (1949)
	Rabbit	i.p	0.95 (UO_2_(NO_3_)_2_·6H_2_O)	Haven and Hodge (1949)
	Guinea pig	iv	0.3 (UO_2_(NO_3_)_2_.6H_2_O)	Haven and Hodge (1949)
	Rat	i.v	1.0 (UO_2_(NO_3_)_2_·6H_2_O)	Haven and Hodge (1949)
	Rat (50–100 g) Rat (150–200 g) Rat (300–400 g)	i.p	60.28 (UO_2_F_2_) 67.23(UO_2_F_2_) 30.91(UO_2_F_2_)	Haven and Hodge (1949)
	Rat (50–100 g) Rat (150–200 g) Rat (300–400 g)	i.p	384.77 (UCl_4_) 271.97 (UCl_4_) 186.11 (UCl_4_)	Haven and Hodge (1949)
	Rat (50–100 g) Rat (200–300 g) Rat (300–400 g)	i.p	144.61 (UO_2_(NO_3_)_2_·6H_2_O) 96.72 (UO_2_(NO_3_)_2_·6H_2_O) 60.69 (UO_2_(NO_3_)_2_·6H_2_O)	Haven and Hodge (1949)
	Rat	i.p	30.91 (UO_2_F_2_) 250.67 (UCl_4_)	Voegtlin and Hodge (1953) (in McDiarmid et al. 2012)
	Rat	p.o	114 (UO_2_(NO_3_)_2_·2H_2_O)	Domingo et al. (1987)
	Mice	i.v	10–20 (UO_2_(NO_3_)_2_·6H_2_O)	Haven and Hodge (1949)
	Mice	i.p	17.37 (UO_2_(NO_3_)_2_·6H_2_O)	Sangeetha Vijayan et al. (2018)
	Mice	p.o	136 (UO_2_(NO_3_)_2_·6H_2_O)	Domingo et al. (1987)
Thorium	Mice	i.v	44.3 (ThCl_4_)	Watanabe (1957)
	mice	i.p	370.8(Th(NO_3_)_4_) 589.1(ThCl_4_)	Syao-Shan (1970)
	Rat	i.p	68 (Th(NO_3_)_4_)	McClinton and Schubert (1948)
	Rat	i.p	648 (24 h) (Th(NO_3_)_4_) 513 (48 h) (Th(NO_3_)_4_)	Downs et al. (1959)

The compounds administered are given in brackets. The LD_50_-values correspond to the amount of uranium (mg U/kg) and not the compound as a whole (data from the literature were adapted if necessary)

*i.v.* intravenous administration, *i.p.* intraperitoneal administration, *p.o*. per os

^a^Assuming mean rabbit weight of 2 kg. For Downs et al. (1959) the mean doses leading to death within 24 h or 48 h are given

Besides experimental data, there is a body of literature on the clinical use and on acute poisonings with uranium compounds that permit to reasonably estimate the acute median lethal dose of uranium (LD_50_) in man. This has been reported with 1000 mg uranium in a soluble compound for inhalation and 5000 mg for ingestion (Kathren and Burklin 2008; Kathren 2009). These values are considered conservative. It is acknowledged that in such acute poisonings, death is related to the chemotoxic properties of uranium on the kidneys. According to the ICRP the absorption quote for uranium in a soluble compound (type "F") after ingestion is given with *f* = 0.02. Thus, the median lethal dose of systemically available uranium can be estimated with 100 mg (= 5000 mg × 0.02), i.e., for a 70 kg body weight adult 1.43 mg/kg.

For thorium, data on acute toxicity are rather sparse compared to uranium, and variable experimental protocols make comparisons difficult (Fig. 3; Table 3). In mice administered thorium chloride intravenously, the mean lethal dose was determined at 44.3 mg thorium/kg, and the tolerated dose at 5 mg/kg (Watanabe 1957). For the same species and intraperitoneal administration, the mean lethal doses for thorium nitrate and thorium chloride were given at 370.8 mg/kg and 589.1 mg/kg, respectively, based on the observation of death cases within 30 days (Kurlyandskaya 1970; Syao-Shan 1970). Values in the same order of magnitude were reported for mature albino rats (body weight. 200 g) given thorium nitrate by intraperitoneal injection: the mean lethal dose was shown to be 648 mg thorium/kg (corresponding to 1520 mg/kg thorium nitrate) within 24 h and 513 mg/kg (1220 mg/kg thorium nitrate) within 48 h (Downs et al. 1959). Weanling albino rats, however (70 g), were more resistant, with lethal doses estimated between 2000 and 2500 mg/kg. On the other side, again, for rats administered thorium nitrate intraperitoneally, much lower mean lethal doses have also been reported (68 mg/kg) (McClinton and Schubert 1948) (Fig. 3). The tolerated amount in this study was given at 48.6 mg/kg (McClinton and Schubert 1948).

High thorium doses with no or only few death cases were reported after oral gavage administrations of thorium nitrate in rabbits (483 mg/kg: no deaths) (Sollman and Brown 1907) and mice (1000 mg/kg: 4 death among 20 animals; 760 mg/kg: no death) (Patrick 1948). The median lethal dose in the latter study was estimated at 1760–2000 mg/kg, and measurements showed that despite thorium nitrate being a soluble compound, most of the amount administered orally was recovered in the feces, so that toxicity was explained by local actions in the guts. A single male mongrel dog who was given thorium nitrate orally 10 g/kg/day that was reduced to 5 g/kg/day after 3 days died the 5th day (Downs et al. 1959). At a dose of 1 g/kg/day over 46 days, another dog survived (Downs et al. 1959).

We could not identify published data on the acute toxicity of thorium in humans that would permit us to reasonably estimate a median lethal dose due to its acute chemotoxicity. In a case report with the suspicion of a possible self-poisoning with thorium, measurements in urine revealed significantly elevated levels for thorium-232 (261 mBq/24-h urine) and thorium-238 (645.3 mBq/24 h) (Razafindranaly and Deschamps 2012). As thorium-238 has a very short physical half-life (*T*_1/2_ = 9 min), we would infer that in case of a suicide attempt, the intake has probably occurred shortly before seeking medical help. However, in the described case, it cannot be excluded that the patient showing psychiatric symptoms voluntarily contaminated his urine sample by soaking thoriated electrodes in it (Razafindranaly and Deschamps 2012), so no reasonable conclusions may be drawn from the case. Thus, to assess acute thorium chemotoxicity, the results of animal experiments remain the primary source of information.

At first sight, the available data suggest that uranium might have a slightly higher acute chemical toxicity than thorium (Fig. 3). However, LD_50_-values, even in the same species, are known to differ between different laboratories, and in particular, for thorium, data are quite sparse and show large variability. The reported lethal doses for uranium and thorium are in the same order of magnitude so the data do not give evidence for substantial differences in acute chemical toxicity.

### Deterministic radiation toxicity: median lethal doses based on radiotoxicity

**Fig. 4:**
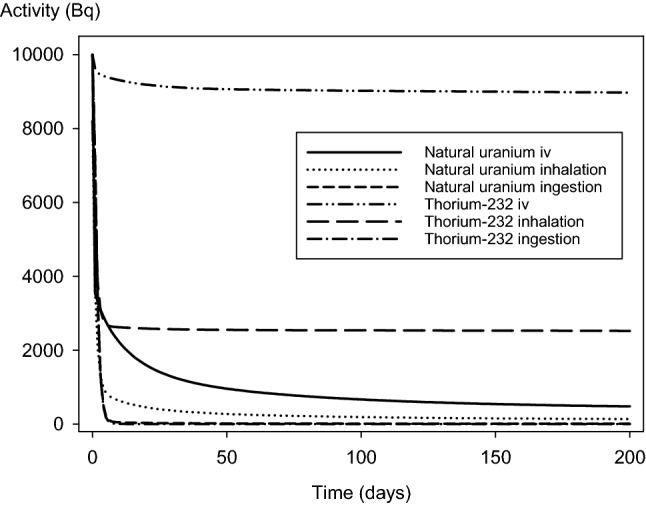
Time-course of the activity of natural uranium or thorium-232 in the whole body after intravenous injection, inhalation, or ingestion of 10,000 Bq. Calculations were done using IMBA software based on the ICRP models for both radionuclides

The fractions of the 50-year dose absorbed within the first 10 days, estimated on the base of the whole-body retention of radioactivity (see Sect. 2.3), are much lower for thorium than for uranium administered as soluble compounds: in the case of injection, 0.094% vs. 9.3%, after inhalation 0.12% vs. 15.5%, and after ingestion 25.9% vs. 74.2%, for thorium and uranium, respectively. For uranium, these fractions are in the same order of magnitude as reported previously (60% after ingestion, 8–9% after inhalation) (Rump et al. 2019). Values nevertheless slightly differ because of the differences in granularity when calculating the area under the curve. The reasons for the differences between thorium and uranium become apparent when viewing the course of the total activity in the body over time, with a sharp drop during the first days in the case of uranium and a much slower protracted activity decrease for thorium (Fig. 4).

**Fig. 5:**
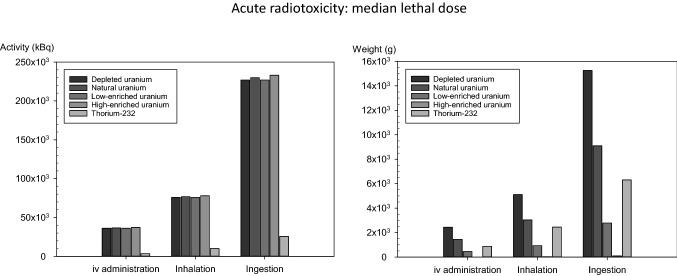
Activities (Bq) or weight amounts (g) of natural uranium or thorium-232 in soluble compounds leading to an equivalent dose of 3.5 Sv absorbed by the red bone marrow (median lethal radiological dose within 60 days without intensive therapy). The values are theoretical, as death is expected to occur due to the chemical toxicity of the metals before reaching lethal radiotoxic amounts

The activities leading to 3.5 Sv equivalent dose to the red bone marrow depend on the route of intake, and for uranium lie in a range of 36 × 10^6^ Bq after intravenous injection, 77 × 10^6^ Bq after inhalation, and 230 × 10^6^ Bq in the case of ingestion (Fig. 5; Table 4). The values are practically independent of the enrichment grade, as the biokinetics depend on the element and not the isotopes. Physical decay half-lives are very long compared to kinetic exchange processes. Small differences may be caused by imprecisions of calculations, as IMBA software gives dose results only with 3 significant digits. Compared to uranium, thorium-232 shows a higher acute radiotoxicity as the red bone marrow equivalent dose corresponding to 50% lethality is attained with lower activities for all intake pathways considered (iv: 3.56 × 10^6^ Bq; inhalation 9.95 × 10^6^ Bq; ingestion: 25.6 × 10^6^ Bq) (Fig. 5; Table 4).

**Table 4:** Quantitative comparison of the radiotoxicity of uranium at different enrichment grades and thorium-232 for soluble and insoluble compounds and different intake pathways.

Spec. act. (Bq/g)	Depleted uranium		Natural uranium		Low enriched uranium		High enriched uranium		Thorium-232	
	14,879		25,270		81,460		2,520,100		4060	
	Bq	g	Bq	g	Bq	g	Bq	g	Bq	g
Deterministic radiotoxicity (LD_50_, RBM dose 3.5 Sv)										
Soluble compounds										
iv admin	36.2 × 10^6^	2432.96	36.6 × 10^6^	1448.40	36.2 × 10^6^	444.39	37.1 × 10^6^	14.72	3.56 × 10^6^	875.60
Inhalation	76.0 × 10^6^	5107.87	76.8 × 10^6^	3039.18	76.0 × 10^6^	932.97	78.0 × 10^6^	30.95	9.95 × 10^6^	2450.70
Ingestion	227 × 10^6^	15,256.00	230 × 10^6^	9101.70	227 × 10^6^	2786.60	233 × 10^6^	92.46	25.6 × 10^6^	6305.40
Stochastic radiotoxicity (critical effective dose 200 mSv)										
Soluble compounds										
iv admin	96,000	6.45	92,500	3.66	89,500	1.10	88,000	0.035	84.5	0.021
Inhalation	336,000	22,58	324,500	12.84	313,000	3.84	308,000	0.12	301	0.074
Ingestion	4.43 × 10^6^	297.74	4.28 × 10^6^	169.4	4.13 × 10^6^	50.70	4.06 × 10^6^	1.61	1.69 × 10^5^	41.63
Poorly soluble compounds										
Inhalation	33,900	2.28	31,900	1.26	30,100	0.370	29,400	0.0117	8100	1.995

Deterministic radiotoxicity: theoretical mean lethal doses (LD50) are defined as amounts leading to the absorption of an equivalent dose of 3.5 Sv by the red bone marrow within the first 10 days after incorporation. Stochastic radiotoxicity: Amounts leading to a committed effective dose (50 years) of 200 mSv defined as critical dose. For poorly soluble compounds, only the inhalative pathway is considered as practically relevant

*Spec. act.* specific activity, *Depl*. depleted, *Nat*. natural, *Low enr.* low enriched, *High enr*. high enriched. *iv admin.* intravenous administration

**Fig. 6:**
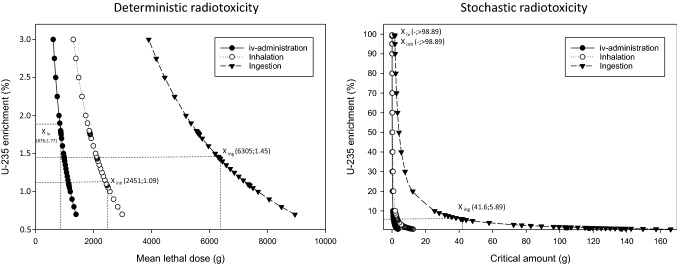
Isoeffective weight-enrichment combinations of uranium leading to 3.5 Sv equivalent dose to the red bone marrow within 10 days (mean lethal dose for radiotoxicity) on the left or to a committed effective dose (50 years) of 200 mSv (defined as critical dose) on the right. The *X*_iv_, *X*_inh,_ and *X*_ing_ show the enrichment grade of uranium required to cause a similar (deterministic or stochastic) radiation damage as the same amount of thorium-232 in weight units (g) depending on the intake pathway (*iv* intravenous administration, *inh*. Inhalation, *ing*. ingestion)

The mean lethal doses for acute radiotoxicity expressed in weight units show another pattern when given in activity units, as values depend on the specific activity of the compound or mixtures (Fig. 5; Table 4). A comparison of thorium-232 and uranium at different enrichment grades show that thorium-232 has a higher deterministic radiotoxicity than natural uranium but is less radiotoxic than low-enriched uranium for all routes of intake considered (Fig. 5, Table 4). The uranium enrichment grades that would lead to the same acute radiotoxicity as thorium-232 numerically depend on the intake pathway but lie all in the range between 1.09 and 1.77% (Fig. 6; and Table 5). The excess or reduction of thorium-232 toxicity relative to uranium at different enrichment grades is shown in Table 6.

**Table 5:** Uranium-235 enrichment grade required to induce a similar deterministic (mean lethal dose, 3.5 Sv absorbed by the red bone marrow within 10) or stochastic radiotoxicity (committed effective dose = 200 mSv) as thorium-232 in the case the same amount in weight units is incorporated through the same intake pathway.

	Deterministic radiotoxicity	Stochastic radiotoxicity
iv administration		
Weight	875.6 g	n.d
U-235 enrichment	1.77%	> 98.9%
Inhalation		
Weight	2,451 g	n.d
U-235 enrichment	1.09%	> 98.9%
Ingestion		
Weight	6,305	41.63 g
U-235 enrichment	1.45%	5.89%

The U-235 enrichment needed is over 98.9%, but the formula used to calculate the weight % of U-234 cannot be meaningfully applied at such enrichment grades

*N.d.* not determined

**Table 6:** Ratio of lethal doses of thorium-232 and uranium in soluble compounds due to acute radiotoxicity (LD_50 Th_/LD_50 U_) and critical amounts of thorium-232 and uranium in soluble compounds leading to a committed effective dose of 200 mSv (*D*_crit. Th_/*D*_crit. U_) depending on the route of intake

	Unit	iv admin	Inhalation	Ingestion
Acute radiotoxicity				
LD_50 Th_/LD_50 Depl. U_	Bq	0.0983	0.131	0.113
	g	0.360	0.480	0.413
LD_50 Th_/LD_50 Nat. U_	Bq	0.0973	0.130	0.111
	g	0.605	0.806	0.693
LD_50 Th_/LD_50 low enr. U_	Bq	0.0983	0.131	0.113
	g	1.970	2.627	2.263
LD_50 Th_/LD_50 high enr. U_	Bq	0.0960	0.128	0.110
	g	59.48	79.18	68.20
Stochastic radiotoxicity				
*D*_Crit. Th_/*D*_Crit. Depl. U_	Bq	0.000880	0.000896	0.0381
	g	0.00326	0.00328	0.1398
*D*_Crit. Th_/*D*_Crit. Nat. U_	Bq	0.000914	0.000928	0.0395
	g	0.00574	0.00576	0.246
*D*_Crit. Th_/*D*_Crit. low enrich. U_	Bq	0.000944	0.000962	0.0409
	g	0.0191	0.0193	0.8211
*D*_Crit. Th_/*D*_Crit. high enrich. U_	Bq	0.00096	0.000977	0.0416
	g	0.600	0.617	25.857

*LD*_*50*_ mean lethal dose, *Depl*. depleted, *Nat*. natural, *enrich*. enriched, *Crit*. critical

**Fig. 7:**
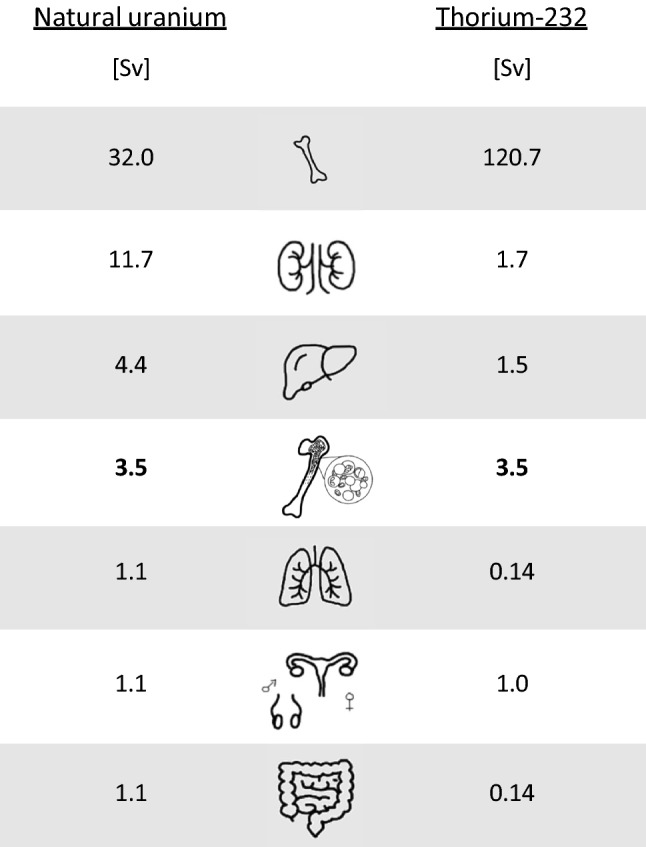
Equivalent doses absorbed by different organs/tissues (bone, kidney, liver, red bone marrow (RBM), lung, gonads, colon) within the first 10 days after the intravenous administration of natural uranium (3.66 × 10^7^ Bq, 1448 g) or thorium-232 (3.56 × 10^6^ Bq, 875.6 g) in activities leading both to an internal irradiation of 3.5 Sv of the RBM (median lethal dose within 60 days without intensive therapy)

The equivalent doses that the different organs and tissues would have absorbed show that for uranium the radiological load of the kidney and liver would be higher than for thorium-232. In contrast, the latter more heavily concentrates in the bone leading to an equivalent dose roughly 4 times larger (Fig. 7).

It must be emphasized that the mean lethal values for acute radiotoxicity must be considered fictive theoretical results (that is why we used the subjunctive in some sentences). The amounts that would be necessary for an equivalent dose of 3.5 Sv being absorbed by the red bone marrow are well above the mean lethal values for the chemotoxicity of uranium, even if enriched, as well as thorium-232 (compare Table 3, 4). So, death by chemotoxicity is to be expected before acute radiation sickness occurs. In addition, except for highly enriched uranium, the amounts of uranium or thorium-232 compounds necessary to induce radiation sickness would be in the range of several hundreds of grams or even kilograms, and from a practical point of view, incorporation at once seems almost impossible.

### Stochastic radiation toxicity: critical uranium and thorium amounts in soluble compounds

**Fig. 8:**
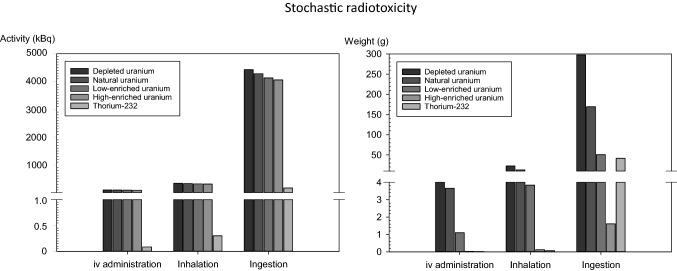
Activities (Bq) or weight amounts (g) of natural uranium or thorium-232 in soluble compounds leading to a committed effective dose (50 years) of 200 mSv defined as critical effective dose. The effective dose is a metric of stochastic radiation damage

Similarly to acute radiotoxicity, the activities of uranium required to lead to a committed effective dose of 200 mSv are comparable for all enrichment grades and only depend on the route of intake (Fig. 8; Table 4). Again thorium-232 appears to be more radiotoxic than uranium when values are expressed in activity units (Fig. 8; Table 4).

The amounts in weight units leading to a committed effective dose of 200 mSv are also shown in Fig. 8 and Table 4. A comparison of thorium-232 and uranium at different enrichment grades shows that thorium-232 has an even higher stochastic radiotoxicity than high-enriched uranium in the case of intravenous administration or inhalation (Figs. 6, 8, Tables 4, 5). Only in the case of ingestion, it is less radiotoxic than high-enriched uranium and just slightly exceeds the toxicity of low enriched mixtures. The excess toxicity of thorium-232 compared to uranium is more pronounced for its stochastic radiation damages than for the (theoretical) deterministic effects (Table 6). Although uranium and thorium-232 amounts were calculated to cause a committed effective dose of 200 mSv, the conclusions on the relative radiotoxicities of both radionuclides can be generalized, as radiological doses behave proportionally to activities.

The uranium enrichment grades that would lead to the same stochastic radiotoxicity as that of thorium-232 expressed in weight units could only be calculated for ingestion (41.6 g uranium enriched to 5.89% would lead to 200 mSv committed effective dose like 41.6 g thorium-232) (Fig. 6; Table 5). In the case of intravenous administration or inhalation, enrichment grades exceeding 98.89% would be needed according to our calculations. The formula used to derive the relative amount of U-234 in the mixture (see “Deterministic radiation toxicity: Quantitative comparison of the theoretical median lethal doses based on radiotoxicity”) cannot be meaningfully applied at such high grades, as the sum of the relative amounts of U-235 and U-234 would exceed 100%.

### Stochastic radiation toxicity of inhaled poorly soluble compounds of thorium and uranium

All previous results relate to the incorporation of soluble thorium-232 or uranium compounds. The critical amounts of thorium-232 or uranium at different enrichment grades inhaled as poorly soluble compounds are displayed in Table 4.

Regarding stochastic radiotoxicity, thorium-232 activity must be increased to reach the same committed effective dose when the solubility of the compound, including the nuclide, decreases (for 200 mSv from 301 Bq in type "F" compound to 8100 Bq in a type "S" compound), i.e., stochastic radiotoxicity is decreasing. In comparison to thorium-232, for uranium the same committed effective dose is obtained with lower activities when the solubility of the compound decreases (for 200 mSv from 336,000 Bq for type "F" to 33,900 Bq for type "S"), i.e., stochastic radiotoxicity is increasing with decreasing compound solubility (Table 4).

Nevertheless, similar to soluble compounds expressed in activity units, the stochastic radiotoxicity of inhaled thorium-232 is more marked than for uranium. But, taking into account the enrichment grade expressed in weight units, the radiotoxicity of thorium-232 ranges between that of depleted and natural uranium (Table 4).

**Fig. 9:**
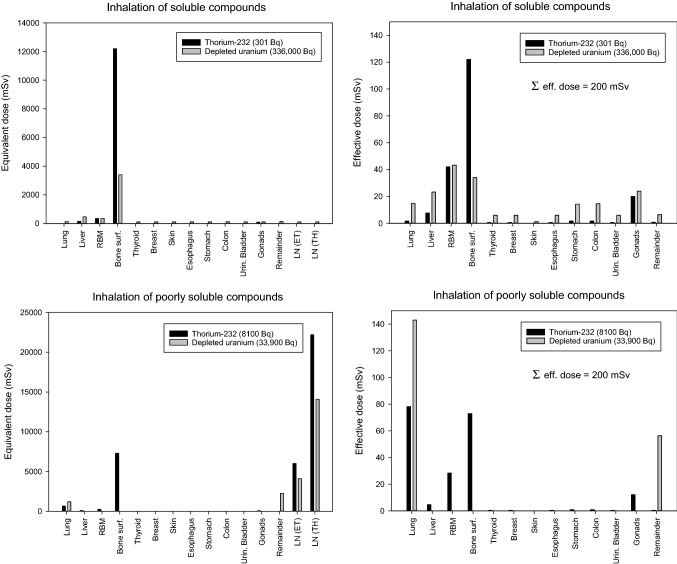
Equivalent and effective doses (equivalent dose × tissue weighting factor for stochastic radiation damages) absorbed by different organs/tissues after inhaling a critical amount of thorium-232 as soluble or poorly soluble compound. Values represent the total dose absorbed over 50 years following incorporation. The critical amount is defined as the activity leading to a total committed effective dose of 200 mSv. *RBM* red bone marrow, *LN (ET)* extrathoracic lymph nodes, *LN (TH)* thoracic lymph nodes. Lymph nodes do not enter into the calculation of the effective dose and thus are not represented in the diagrams of the effective doses on the right

Even in the case of inhalation of a poorly soluble thorium-232 compound, the bone surfaces absorb an exceptionally high equivalent and effective dose beside the conducting airway and lung, including the lymph nodes (Fig. 9, Tables 7, 8). The equivalent dose absorbed by the thoracic lymph nodes is even higher than for the bone surface (22,200 mSv after the inhalation of 8100 Bq vs. 7000 mSv for the bone surface). Equivalent doses absorbed by the lymph nodes, however, do not enter into the calculation of the effective dose, as they are not included in the remainder tissues. Although this is not a realistic scenario, assuming that the whole radioactive load of a Milan anti-tank missile (10 kBq) would acutely be inhaled as poorly soluble oxides, this would result in a committed effective dose of 247 mSv (Table 8).

**Table 7:** Equivalent and effective doses absorbed by different organs/tissues over 50 years following the inhalation of soluble (type “F”, fast absorption) or poorly soluble (type “S”, slow) thorium-232 compounds or depleted uranium at activities leading to a committed effective dose of 200 mSv

	Tissue weight	Thorium-232				Depleted uranium			
		Soluble compound (F) (301 Bq, 0.0741 g)		Unsoluble compound (S) (8100 Bq, 1.995 g)		Soluble compound (F) (336,000 Bq, 22.58 g)		Unsoluble compound (S) (33,900 Bq, 2.278 g)	
		Equ. dose (mSv)	Eff. dose (mSv)	Equ. dose (mSv)	Eff. dose (mSv)	Equ. dose (mSv)	Eff. dose (mSv)	Equ. dose (mSv)	Eff. dose (mSv)
Lung	0.12	13.6	1.64	652	78.2	122	14.7	1190	143
Liver	0.05	152	7.61	94.4	4.72	466	23.3	1.16	0.058
RBM	0.12	350	42	236	28.3	360	43.3	0.894	0.107
Bone surf	0.01	12,200	122	7300	73.0	3400	34.0	8.37	0.0837
Thyroid	0.05	13.6	0.681	7.99	0.399	119	5.97	0.275	0.0138
Breast	0.05	13.6	0.679	8.0	0.400	119	5.97	0.281	0.0140
Skin	0.01	13.6	0.136	7.96	0.0796	119	1.19	0.274	0.00274
Esophagus	0.05	13.6	0.680	8.02	0.401	119	5.97	0.282	0.0141
Stomach	0.12	13.6	1.63	7.98	0.958	119	14.3	0.294	0.0353
Colon	0.12	13.6	1.64	8.08	0.97	121	14.6	0.769	0.0923
Urine Bl	0.05	13.6	0.680	7.94	0.397	120	5.99	0.274	0.0137
Gonads	0.2	99.9	20	60.7	12.1	119	23.9	0.273	0.0545
Remainder	0.05	15.2	0.758	9.41	0.471	131	6.54	2260 + 0.306^a^	56.4^a^
LN (ET)	–	13.6	–	6010	–	119	–	4090	–
LN (TH)	–	13.6	–	22,200	–	119	–	14,100	–
Sum	1.0	–	200	–	200	–	200	–	200

Equ. dose: equivalent dose; Eff. Dose: effective dose (= equivalent dose × tissue weighting factor from ICRP 68). For thorium-232 compounds and soluble uranium compounds, the bone surface is a particularly important contributor to the effective dose. Remainder tissues: muscles, brain, small intestine wall, kidneys, pancreas, spleen, thymus, uterus, adrenals, extrathoracic airways (ET) (included by IMBA)

^a^As the equivalent dose absorbed by a remainder tissue (here ET 2260 mSv) is larger than the dose absorbed by any other tissue, this largest dose and the mass-weighted mean equivalent dose of the other remainder tissues (0.306 mSv) are each multiplied by 0.025 to calculate the effective dose (“splitting rule”). LN (ET): extrathoracic lymph nodes; LN (TH): thoracic lymph nodes. Equivalent doses absorbed by the lymph nodes do not enter into the calculation of the effective dose

**Table 8:** Equivalent and effective doses resulting from the inhalation of the total radioactive load of a MILAN anti-tank missile (10 kBq thorium-232) (purely thought experiment, not a realistic scenario)

Organ/tissue	Equ. dose (mSv)	Eff. dose (mSv)	Organ/tissue	Equ. dose (mSv)	Eff. dose (mSv)
RBM	291	35.0	Adrenals	9.96	Remainder
Bone surf	9010	90.1	Brain	9.87	Remainder
Lung	804	96.5	Kidneys	132	Remainder
Liver	117	5.83	Muscle	9.86	Remainder
Thyroid	9.86	0.493	Pancreas	9.89	Remainder
Breast	9.87	0.494	Small int	9.85	Remainder
Skin	9.83	0.0983	Spleen	9.87	Remainder
Urinary bladder	9.81	0.490	Thymus	9.90	Remainder
Esophagus	9.90	0.495	Uterus	9.82	Remainder
Stomach	9.85	1.18	ET	1080	Remainder
Upper low int	9.91	Incl. in colon	Total Remainder	11.6^c^	0.581
Lower low int	10.1	Incl. in colon			
Colon^a^	9.98	1.20	LN (ET)^d^	7420	–
Testes	74.9	Incl. in gonads	LN (TH)^d^	27,400	–
Ovaries	73.5	Incl. in gonads			
Gonads^b^	74.9	15			
Committed Eff. Dose (= sum eff. doses in column 3 + total Remainder in column 6) = 247.46 mSv					

*Equ. Dose* equivalent dose, *Eff. dose* effective dose, *Bone surf* bone surface, *int.* intestine, *ET* extrathoracic airway, *incl*. included

^a^The colon dose is the mass-weighted average of the upper and lower large intestine equivalent doses

^b^The gonads dose is the highest of testes and ovaries equivalent doses

^c^The remainder dose is the mass-weighted average equivalent dose for the remainder tissues

^d^Equivalent doses absorbed by the lymph nodes do not enter into the calculation of the effective dose

*LN (ET)* extrathoracic lymph nodes, *LN (TH)* thoracic lymph nodes

After inhalation of a poorly soluble uranium compound, the lungs and the airway are tissues that absorb particularly high doses. The extrathoracic airway, that belongs to the remaining tissues in the default settings of IMBA, absorbs the highest equivalent dose (2260 mSv for 33,900 Bq depleted uranium), among all other tissues that are included for the calculation of the total effective dose (Fig. 9; Table 7). The tissues absorbing even higher equivalent doses, but that are not taken into account for the calculation of the effective dose, are again the extrathoracic and thoracic lymph nodes (4090 and 14,900 mSv, respectively). It must be mentioned that the doses absorbed by these tissues depend on the deposition pattern in the airway and, thus, the size of the particles (in our calculations, we used an AMAD of 5 µm, see “The biokinetic-dosimetric models used for radiological dose assessments after uranium or thorium incorporation”). In comparison to thorium-232, the radiological load of other internal organs (e.g., bone surfaces, red bone marrow) is much lower (Fig. 9; Table 7).

## Discussion

The quantification of acute toxicity of a chemical is a fundamental toxicological investigation. For poisons showing a threshold value, dose–response curves permit to derive as metrics the No-observed-effect-level (NOAEL), the Lowest-observed-effect-level (LOAEL), and the median lethal dose (LD_50_) (US-EPA 2021). The mean lethal dose has the virtue to be simple to estimate. However, it is influenced by many intrinsic and extrinsic factors (species, strain, age, weight, cage population, feed and water quality, nutritional state, etc.). It, thus, shows a large inter-laboratory variability, even when study designs seem comparable (Gad and Chengelis 1998). It has also been acknowledged for a long time that approximate lethal doses with comprehensive studies of the pathophysiological mechanisms of the toxic effects provide more important information for emergency physicians than precise LD_50_-values alone (Zbinden and Flury-Roversi 1981). Nevertheless, LD_50_-values are convenient metrics for classification and comparing the acute toxicity of two substances. That is the reason why we focused on these metrics to assess the chemotoxicity of uranium and thorium, besides the availability of these values in older publications. However, conclusions from lethal doses in animal experiments about the toxicity in humans are to be drawn very cautiously.

In addition to direct toxic mechanisms on tissues and metabolism, the kind of administration and the associated differences in systemic bioavailability must always be considered. Intraperitoneal injections in pre-clinical studies are often considered comparable to intravenous administrations. This is, however, dubious, and it was even suggested that the concentration time course is rather comparable to an oral administration. In peritoneal dialysis patients, the pharmacokinetics of erythropoietin administered intraperitoneally was investigated. It was shown that the maximum serum concentrations were similar after intraperitoneal and subcutaneous administration, but amounted only to 5% of the value after intravenous injection (Ateshkadi et al. 1993). In the case of thorium, it was shown that this tetravalent ion is heavily hydrolyzed at pH-values over 3.5 (i.e., at physiological pH), and the hydroxides form chain-like complexes are poorly soluble in water. This has been given as an explanation for its poor gastrointestinal absorption and its poor bioavailability after intraperitoneal injection, as a large part will stay at the site of administration (Traikovich 1970). Assuming that the acute chemotoxicity of thorium is concentration dependent, this would possibly explain that the median lethal doses for soluble thorium compounds after intraperitoneal and oral administrations are in part in a similar order of magnitude of several hundred or even exceeding 1000 mg/kg and much higher than after intravenous injection (44.3 mg/kg in mice) (Watanabe 1957).

At first sight, the impression could arise that the chemotoxicity of uranium is somewhat more pronounced than that of thorium when considering experimental findings after intravenous or intraperitoneal injections (Fig. 3). This would be in line with an estimation that thorium is about 7 times less toxic than uranium when administered as soluble salts (Downs et al. 1959).

Uranium compounds have been described as "feeble poisons" (Hodge et al. 1973; Kathren 2009), and viewing the data, the same should therefore apply to thorium. But a comparison with the toxicity of other metals is interesting. The lethal oral doses for iron sulfate have been given with about 200 mg/kg (74 mg iron/kg), for thallium sulfate with 14 mg/kg (9.5 mg thallium/kg) or for arsenic with 4.3 mg/kg (3.25 mg arsen/kg) (Ludewig and Lohs 1991; Schäfer and Maurer 1993; Reichl 1997). Taking into account bioavailabilities, these values correspond roughly to 7.4 mg/kg for iron becoming systemically available (assuming a bioavailability of 10% with a very large variability) (Farrag et al. 2019), 7.6 mg/kg for thallium and 2.6 mg/kg for arsen (80% bioavailability for both metals) (Reichl 1997). The lethal amount of systemically available uranium in man can be estimated at 1.43 mg/kg (5000 mg/70 kg = 71.4 mg/kg per os; 71.4 mg/kg × 0.02 = 1.43 mg/kg). For thorium injected intravenously, the mean lethal dose in mice was given with 44.3 mg/kg (Watanabe 1957). For thorium, the gastrointestinal absorption fractions are lower than for uranium and have been reported in a range of 0.001 (0.1%) to 0.01 (1%) (Burkart 1984; Johnson and Lamothe 1989) (absorption quote given by the ICRP for soluble thorium compounds *f* = 0.005) (ICRP 1995b). Applying the ICRP value to the estimated median lethal dose in mice in case of oral administration (1760–2000 mg/kg) (Patrick 1948) would result in a lethal dose of 10 mg/kg (= 2000 mg × 0.005) being available systemically. These values suggest that compared to iron, thallium or arsen, it is not justified to generalize that uranium and thorium are “feeble poisons”, as systemically they are quite toxic. Their apparent toxic mildness seems to result from their pharmacokinetic properties and very low systemic bioavailability.

The median lethal values for uranium and thorium also do not permit drawing conclusions on the mechanism of their toxic action. In blood, uranium is transported as soluble uranyl carbonate ion (UO_2_^2+^, hexavalent uranium) (Durbin 1984; McDiarmid et al. 2012) that has a sufficiently low molecular weight to be filterable at the glomerulus (Stevens et al. 1980; Durbin 1984; McDiarmid et al. 2012) and the main target organ of its chemical toxicity is the kidney (Kathren 2009). Increased urinary excretion of electrolytes, proteins (albumin, β-2-microglobulin), and enzyme activities (N-acetyl glucosaminidase, alkaline phosphatase) indicate tubular lesions, particularly of the proximal tubules after uranium exposure. At the subcellular level, it was reported that uranium affects the solute transport through the tubular cell membranes and inhibits mitochondrial oxidative phosphorylation (Brady et al. 1989). Like many metals that are not shielded, uranium may induce the generation of reactive oxygen species (ROS) that are chemically highly reactive (Gagandeep et al. 2014), which may subsequently impair numerous cell functions. A reduction of the glomerular filtration rate was described (increase of plasma creatinine and ureic nitrogen), but it is unknown whether this is caused by a direct toxic effect on the glomerulus or if it is rather an indirect effect of the impairment of the tubular function (tubuloglomerular feedback) (Vicente-Vicente et al. 2010).

The toxic mechanism of thorium and its chemical interaction with tissue constituents are less well documented. Thorium is present in blood mainly in its tetravalent form (Paquet 2014), and its charge/size ratio differs from hexavalent uranium and is more similar to trivalent iron (Kumar et al. 2013). It is transported bound to transferrin (Peter and Lehmann 1981; Kumar et al. 2013), although it is not known whether the binding site is the same as for iron, and it has an organ distribution profile similar to plutonium, with most accumulating in the bone, the liver and the spleen (Leiterer et al. 2010; Kumar et al. 2013). After chronic injections in mice, alterations of liver function, as reflected by an increase of alanine aminotransferase (ALT) and alkaline phosphatases (AP) in serum were observed (Kumar et al. 2008). Moreover, the activities of superoxide dismutase (SOD) and catalases (CAT) were decreased in several tissues, particularly in the liver, and lipid peroxidation markers were enhanced, suggesting an accumulation of reactive oxygen species. These biochemical alterations were associated with histological modifications of liver tissue (Kumar et al. 2008). As known for many other metals, including uranium, it seems that through its catalytic activity, thorium may contribute to the generation of free radicals (Fenton chemistry) and lead to oxidative stress (Mizukami-Murata et al. 2006), indirectly affecting numerous physiological functions with the liver being an important target organ of toxicity. Further studies have shown that oxidative stress is also induced by thorium in the central nervous system of mice, as reflected by decreases in antioxidant enzyme activities, oxidation markers, and modifications of cholinergic functions as well as neurobehavioral impairments (Kumar et al. 2009).

A comparison of the radiotoxicity of two radionuclides is much more challenging than comparing their chemical toxicity. Threshold values for the induction of acute radiation sickness and estimations of median lethal radiological doses assume homogeneous whole-body irradiation within a short time frame. As far as we know, there is no established definition of what is exactly an acute radiation exposure, especially when considering internal uptake of radionuclides. Our calculations are based on the equivalent dose absorbed by the red bone marrow within the first 10 days after incorporation, as previously used in a study to assess deterministic radiation damages of uranium at different enrichment grades (Rump et al. 2019). Exposure time to the initial radiation in the nuclear bomb victims in Hiroshima and Nagasaki was certainly much shorter. On the other side, on the occasion of the Castle Bravo nuclear test on the Marshall Islands in 1954, the residents of the Rongelap Atoll were exposed to fallout for roughly 3 days (Simon et al. 2010; Rump et al. 2022), and the crew of the Japanese fishing boat Lucky Dragon that was in the vicinity of the test site was exposed for 14 days until they returned to their home port in Japan (with probably half the radiological dose absorbed on the first day) (Kumatori et al. 1965; Rump et al. 2022). These radiation exposure patterns over several days were nevertheless suited to induce an acute radiation syndrome in both groups of victims, and this time range is quite comparable to the period of 10 days we used to compute the total radiological dose absorbed by the red bone marrow to assess the possibility of acute radiation sickness induction.

Apart from different intake pathways and bioavailabilities, different reference units, activities (Bq), or weight units (mg), can be used to describe radiotoxicity. In the case of differently enriched nuclide mixtures (e.g., uranium mixtures), the value of the metrics for the acute radiotoxicity will be very similar to identical for all enrichment levels of the mixture if activity units (Bq) are used, since the biokinetics of the isotopes of the same element are identical. This explains the small differences between the acute radiotoxicity of uranium at different enrichment grades when given the activity necessary to achieve a dose of 3.5 Sv in the red bone marrow within the first 10 days (i.e., the mean lethal dose) (Fig. 5 on the left). Mathematical rounding effects could contribute to the tiny fluctuations in the values since the IMBA software gives results only with 3 significant digits. However, differences in the acute radiotoxicity of differently enriched uranium mixtures become very clear if the median lethal dose is not given as an activity but in mass units (Fig. 5, on the right). The reference unit's importance also becomes evident when comparing the acute radiotoxicity of uranium mixtures and thorium-232. Expressed as an activity, thorium-232 is definitely more radiotoxic than uranium mixtures (Fig. 5 on the left), whereas a much more differentiated picture arises when using weight units (Fig. 5 on the right). In the latter case, thorium-232 toxicity can be located between the radiotoxicity of natural and low-enriched uranium (more precisely, its radiotoxicity would correspond to a U-235 enrichment grade of 1.09% to 1.77%, depending on the route of intake). Besides the different biokinetic properties that may explain the higher acute radiotoxicity of thorium-232 expressed as an activity, the lower specific activity of thorium-232 will tend to enhance the mean lethal dose in mass units, suggesting less significant toxicity.

It must be emphasized that the mean lethal doses by acute radiotoxicity are purely theoretical values for uranium and thorium-232. According to our results, the amounts that would be required to cause death by an acute radiation syndrome by far exceed the mean lethal doses due to the chemical toxicity of both metals, and thus acute radiation sickness by incorporation of uranium or thorium-232 is not to be expected in a real clinical setting. However, caution is necessary in the case of enriched uranium. Taking into account the uncertainties of the model and the large variability of the kinetic parameters, it was previously shown that the occurrence of an acute radiation sickness with a hematopoietic syndrome in addition to a life-threatening poisoning caused by its chemotoxicity could not be excluded after ingestion of a soluble compound of highly enriched uranium in particularly sensitive individuals (Rump et al. 2019). Moreover, inhaling a poorly soluble compound of highly enriched uranium in a range corresponding to the mean lethal dose by its chemotoxicity may be expected to cause severe pneumonitis (Rump et al. 2019). Although less attention has been given to the acute toxicity of thorium, and there are fewer data available, our findings clearly confirm the view that "the chemical toxicity of thorium is the limiting factor for organ burdens" (Burkart 1984) and similar to uranium, the acute radiotoxic effects are definitely not a core issue.

For comprehensive statements on the radiotoxicity of a nuclide, besides the deterministic effects, the stochastic radiation damages which lead to health impairments, in the long run, must be considered. This includes not only the induction of malignant tumors (Preston et al. 2003; Richardson et al. 2009), but also the development of other chronic diseases of the circulatory system, the respiratory organs, or the gastrointestinal tract (Wong et al. 1993; Shimizu et al. 1999; Ozasa et al. 2012). In practice, e.g., in occupational medicine, the stochastic radiation effects are the focus of interest, especially since the incorporation of radionuclides, apart from exceptions such as in the Litvinenko case (Roessler 2007), only rarely lead to deterministic effects such as acute radiation sickness (Weickhardt 2001). Some dosimetric differences between deterministic and stochastic radiation damages must be considered.

Besides the absorbed energy dose and the dose rate, the biological effects will depend on the pattern of ionizing energy deposition along the track of the particles. Therefore, the type of radiation (alpha, beta, or gamma) must be taken into account. This is done by defining a relative biological effectiveness (RBE) that is calculated as the energy dose absorbed from a reference radiation divided by the energy dose of a test radiation (alpha-, beta-, neutron-, photon-radiation) producing the same biological effect. A well-known radiation quality factor of 20 has been determined for alpha-radiation, reflecting the relative biological effectiveness compared to gamma- or beta-radiation (ICRP 2007). However, this relates to stochastic radiation damages (cancer risks). For deterministic radiation damages, this value must not be used. Based on cell killing effectiveness, values between 3 and 5 for alpha particles have been described (Sgouros et al. 2010), and a radiation quality factor of 5 has been proposed (Lassman and Nosske 2013). As uranium and thorium-232 are mainly alpha-emitters, that is the value we applied to calculate the mean lethal amounts due to acute radiotoxicity (deterministic damages). This leads to lower, i.e., more conservative lethal doses (expressed in Bq or g), than if using a quality factor of 20, and avoids underestimating deterministic radiotoxicity.

When comparing radiotoxicity based on stochastic radiation damages, we calculated committed effective doses (50 years) with the widely accepted radiation quality factor of 20 for alpha-radiation (ICRP 2007) and the tissue weighting factors for the different organs and tissues as given by the ICRP (1991, 1994) and implemented in IMBA software. This permits quantifying the radiological burden to the whole body with a single dose value that can be related to a loss of statistical lifetime (0.4 day/mSv, averaged over the genders and all age groups) (Oka 2012; Rump et al. 2018a, b). Since there is no scientifically justifiable threshold value below which the health risk from irradiation tends to zero, the limit values specified in regulations on radiation protection for occupational settings are normative in nature, e.g., 20 mSv in European countries or 50 mSv in the US. In the event of radioactivity incorporation after an attack with a dirty bomb, a committed effective dose of 200 mSv is usually considered a clear indication for decorporation treatment in adults if the radionuclide is prone to such therapy (Ménétrier et al. 2005; Rump et al. 2016, 2018b). That is why we used the latter value as the basis for our calculations to compare the stochastic radiotoxicity of uranium and thorium-232. Since there is a proportionality between the incorporated activity and the effective dose, the determined ratios of radiotoxicity are valid independent of the effective dose used for the calculations. It must, however, be emphasized that dose values resulting from radionuclide incorporation should always be considered as estimates, as dose coefficients (determined by the biokinetic models with their parameters, the values of the energy doses absorbed by the organs as well as the tissue weighting factors) evolve over time (e.g., committed effective dose coefficient for thorium-232 in a compound type M: 2.9 × 10^–5^ Sv/Bq, type S: 1.2 × 10^–5^ Sv/Bq in ICRP Publication 119, ICRP 2012; type M: 8.2 × 10^–6^ Sv/Bq, type S: 5.4 × 10^–5^ Sv/Bq in ICRP Publication 137, ICRP 2017).

Similarly to acute deterministic damages, the stochastic radiotoxicity of uranium mixtures at different enrichment grades is quite similar when expressed in activity units but markedly differs when using weight units. Thorium-232 is clearly more radiotoxic than uranium using activity units. The higher radiotoxicity of soluble thorium-232 is even more evident for the stochastic effects than for the deterministic effects. This is not really surprising, as the committed effective dose is calculated for an extended period of 50 years, with thorium-232 being eliminated from the body more slowly so that total body activities remain at a higher level for a long time. In contrast, there is a sharp drop in the uranium activity occurring within a few days (see Fig. 4). Expressed in weight units, the stochastic radiotoxicity of thorium-232 in a soluble compound is also more toxic than uranium, even at a high enrichment grade, except for ingestion. In the latter case, it is just slightly more toxic than low-enriched uranium.

Despite modifications of numerical values over time, the much higher stochastic toxicity of thorium-232 compared to uranium is also reflected by the lower annual limits of intake (Belbéoch 1993; NRC 2021). In the Code of Federal Regulations (CFR 2022), the annual limits of intake (ALI) for occupational exposures (annual dose limit 50 mSv) are given with 10^–3^ µCi (37 Bq) for thorium-232 and 8 × 10^–1^ µCi (29,600 Bq) for uranium-235 and 238 (type W compounds with a clearance half-time between 10 and 100 days from the lungs). Thus, the ALI for thorium-232 is even lower than for plutonium-239 (6 × 10^–3^ µCi, 222 Bq). Although this appears surprising at first glance, the higher stochastic radiotoxicity of thorium-232 compared to plutonium-239 is well known in the literature (Belbéoch 1993; Laroche and Gerasimo 2000). For the sake of completeness, it must be mentioned that implementing the ALI values given above with IMBA software does not result in committed effective doses of 50 mSv. However, results are in the same order of magnitude. The differences in numerical values cannot be corrected by setting the particle sizes from 5 µm (default value used by IMBA) to 1 µm, as assumed for the ALI values above. Rather, the differences are probably due to different respiratory tract models underlying the dose coefficients used by IMBA (respiratory tract model from ICRP Publication 66; ICRP 1994a) and the US NRC Regulation 10 CFR (model from ICRP Publication 30; ICRP 1979).

In comparison to soluble compounds, the stochastic radiotoxicity of thorium-232 inhaled as a poorly soluble compound and expressed in weight units can be located between depleted and natural uranium. This shows that the results of radiotoxicity comparisons are a function of a complex combination involving the nature of the radionuclide with its physical decay characteristics, its systemic biokinetics, the route of intake, and the physicochemical properties of the chemical compound in which the nuclide is integrated. Therefore, regarding the danger emanating from the incorporation of thorium-232 included in MILAN missiles, it seems rather very limited, considering the activity in a single weapon system and the expected release of insoluble oxides. It is nevertheless evident that the contamination of a combat zone or training area will depend on the number of missiles that have been shot.

## Conclusion

On the bottom line, our results do not permit a founded definitive statement on the relative acute chemotoxicity of uranium versus thorium. Conclusions must be done cautiously, as acute toxicity assessments for thorium relies only on experimental findings, and human data are lacking. But it may be stated that for both heavy metals, the acute chemotoxicity clearly exceeds their acute radiotoxicity. Although threshold levels for deterministic radiation damages are reached by lower activities of thorium-232, acute radiation sickness should not be considered a real clinical issue, as toxic effects caused by chemical mechanisms will occur far before the realization of deterministic radiation damages. Concerning the risk of long-term health effects from stochastic radiation damages, our findings confirm that thorium-232 is more radiotoxic than uranium if incorporating the same activities. Using weight units for comparison shows that relative toxicities depend on the enrichment grade of uranium, i.e., the specific activity of the compound. This demonstrates that comparisons of radiotoxicity must always consider the reference units, activity or weight, and it seems meaningful to express results using both units to avoid the arising of misconceptions. Moreover, the physicochemical properties of the compounds integrating the radionuclide and the route of intake are of major importance.

## Data Availability

All the results of our study are based on data freely available in the literature.

## Abbreviations

ALI: Annual limit of intake
AMAD: Activity median aerodynamic diameter
AUC: Area under the curve
Bq: Becquerel
Ci: Curie
DU: Depleted uranium
Gy: Gray
ICRP: International Commission on Radiological Protection
IMBA: Integrated modules for bioassay analysis
LD_50_: Median lethal dose
LOAEL: Lowest-observed-adverse-effect-level
Milan: Missile d’infanterie léger antichar
NOAEL: No-observed-adverse-effect-level
Sv: Sievert
Th: Thorium
U: Uranium
